# Trajectories of mobility difficulty and falls in community-dwelling adults aged 50 + in Taiwan from 2003 to 2015

**DOI:** 10.1186/s12877-022-03613-3

**Published:** 2022-11-25

**Authors:** Fang-Lin Kuo, Chia-Ming Yen, Hung-Ju Chen, Zih-Yong Liao, Yen Lee

**Affiliations:** 1 National Center for Geriatrics and Welfare Research, National Health Research Institutes, Taiwan, 8, Xuefu W. Rd., Huwei Township, Yunlin County 63247 Taiwan; 2 China Medical University, Taichung City, Taiwan; 3 Edgewood College, Madison, WI USA

**Keywords:** Mobility, Falls, Community, Older adults

## Abstract

**Background:**

A decline in mobility leads to fall occurrence and poorer performance in instrumental activities of daily living, which are widely proved to be associated with older adults' health-related quality of life. To inform potential predicaments faced by older adults at different age levels, predictors of this mobility change and falls along with the ageing process need to be further evaluated. Therefore, this study examined the risk factors associated with the longitudinal course of mobility difficulty and falls among community-dwelling middle-aged and older adults in the Taiwanese community.

**Methods:**

We evaluated data for the period between 2003 and 2015 from the Taiwan Longitudinal Study on Aging; the data cover 5267 community-based middle-aged and older adults with approximately 12 years of follow-up. In terms of mobility, the participants self-reported difficulties in mobility tasks (eg, ambulation) and whether they used a walking device. We employed linear mixed-effects regression models and cumulative logit models to examine whether personal characteristics are associated with mobility difficulty and falls.

**Results:**

Mobility difficulty significantly increased over time for the participants aged ≥ 60 years. Perceived difficulties in standing, walking, squatting, and running became apparent from a younger age than limitations with hand function. The probability of repeated falls increased significantly with older age at 70 (*p* = .002), higher level of mobility difficulty (*p* < .0001), lower cognitive status (*p* = .001), living alone (*p* = .001), higher number of comorbid illnesses (*p* < .001), walking device use (*p* = .003), longer time in physical activities (*p* < .011), and elevated depressive symptoms (*p* = .006). Although walking aid use increased the probability of falls, individuals with mobility difficulty had a reduced probability of repeated falls when using a walking device (*p* = .02).

**Conclusion:**

Community-dwelling Taiwanese adults face an earlier mobility difficulty starting in 60 years old. Individuals with more leisure and physical activities in daily life were more likely to maintain mobility and walking safety. Long-term, regular, social, and physical activity could be a referral option for falls prevention program. The use of a walking device and safety precautions are warranted, particularly for individuals with walking difficulties.

**Supplementary Information:**

The online version contains supplementary material available at 10.1186/s12877-022-03613-3.

## Introduction

Older adults with adequate mobility and overall functional independence are considered to have more security and a greater sense of well-being [1]. Studies [2, 3] have revealed that impaired cognition and mobility critically affect older adults’ health-related quality of life. Mobility declines and cognitive impairment often coexist and appear to have a bidirectional temporal relationship. Older adults with faster subjective cognitive decline and cognitive impairment tended to take fewer daily steps and have more mobility problems [4, 5]. Individuals with poorer gait performance than their peers may have greater cognitive decline after 10 years [6]. Moreover, the rate of decrease in gait speed was reported to differ significantly between older adults who developed mild cognitive impairment and those who did not [7]. Similar results were also found in the Health, Aging, and Body Composition Study [8], which indicated that baseline lower executive functions predicted subsequent declines in gait speed. Because both impaired mobility and impaired cognition result in loss of functional independence, thus increasing the risk of institutionalization and mortality, investigating the trajectories of mobility difficulty and fall among community-dwelling older adults is imperative. Understanding the key determinant of mobility difficulty and falls may inform further intervention strategies aimed at combating falls, thus maintaining or improving the functional capacity of community-dwelling older adults.

Participation with leisure and physical activities could be a determinant to middle and older adults. Several findings from studies support that individuals with adequate physical and leisure activities in daily life may gave: reduced risk of mortality [9], lower mobility difficulties [10], and better quality of life [11]. However, these studies did not comprehensively measure the mobility characteristic and did not explore longitudinal changes in the impact of leisure and physical activities in fall occurrence.

Falling is a global gerontological concern, with up to 35% of older adults reporting at least one fall annually; these falls can result in severe injuries, loss of independence, greater disability, and even death [12–15]. The consequences of falls include not only physical injuries but also fear of falls, depression, and need for advanced or long-term health care services [15, 16]. Approximately 20% to 33% of fallers sought medical services with a high prevalence of fractures, and more than 20% of those who were admitted were unable to return home at discharge [12, 13]. The consequences of older adults’ falls represent a substantial burden to health care services. The cost of treating each fall event was estimated to be approximately US$2100 per person in Scotland [12] and US$6479 to US$10,499 in Australia [15].

Fall occurrence has been suggested to predict poorer performance in instrumental activities of daily living as well as gait problems a year after the fall [17, 18]. Risk factors for falls in older adults are multifaceted and involve personal, medical, social, and environmental factors. Impaired gait and balance, being homebound, older age, female sex, diseases such as stroke and arthritis, use of high-risk medication, larger waist circumference, depressive symptoms, sensory problems, history of falls, physical environment, and walking device use have been associated with a higher risk of falls [14, 19–23]. In Taiwan, fall incidence ranges from 19 to 45% in older adults [17, 19, 22, 24], and is drastically elevated in those aged ≥ 70 years [17]. Fear of falling is a common result of fall experiences and frailty [17].

Walking aids, such as canes and walkers, are commonly used by those with walking limitations. Although the use of such devices helps individuals to accomplish difficult activities and increases their participation in activities [25], it poses some safety concerns. Compared with nonusers, those who use a walking device tend to have more falls [21, 22, 26, 27]. In one study, approximately 22% of older adults using assistive devices reported falls [27]. However, many falls are preventable. Use of walking aids is associated with greater fear of falling and a more conservative gait pattern [27]. Bertrand, Raymond, Miller, Ginis, and Demers [25] argued that the benefits of walking aids were determined by the user’s ability to integrate usage of the device into daily life to overcome obstacles. Although walking is generally promoted in functional training for the older population, fall prevention is critical to maintain older adults’ walking safety [20]. Hence, in this study, we examined the personal factors associated with mobility difficulty, falls, and walking device use over time in the middle-aged and older population in Taiwan by using a national database.

## Methods

### Study design

Data for this study were collected from the Taiwan Longitudinal Study on Aging (TLSA), which examined healthy aging and longevity in participants aged ≥ 50 years. The TLSA was initiated in 1989 using stratified, multistage national probability sampling [28, 29], with a longitudinal and cross-sectional data set collected every 3 to 4 years. Informed consent was obtained from the participants. The present study used the data set from 2003 to 2015 with a total of 5267 community-dwelling participants. The protocol of this study was approved by the Research Ethics Committee of the National Health Research Institutes, Taiwan (EC1110104-E).

### Visits

Baseline data were obtained from all participants in 2003, and they were subsequently interviewed at approximately 4-year intervals over a 12-year period (total of 4 study visits). Please see Appendix A for the construction of TLSA sample. Of the 5267 participants for which baselines data were obtained in 2003, 4330, 3579, and 2890 successfully completed the interviews in 2007, 2011, and 2015, respectively.

### Measurements

The participants underwent a comprehensive interview to answer the survey questions. The cognitive status of participants was assessed using 9 items of the Short Portable Mental Status Questionnaire (SPMSQ) and 3 sets of recall questions. Mobility was recorded using a self-reported scale addressing the difficulties in performing 9 mobility tasks. General health status was considered by collecting the following data: the number of comorbid illnesses, walking device use, depressive symptoms surveyed using the Center for Epidemiological Studies-Depression (CES-D), living status, body mass index (BMI), the number of falls within 1 year, and visual and hearing impairments.

#### Demographics and health information

Data on age group, sex, walking device use, perceived mobility difficulty, body weight and height, the number of comorbid illness, and fall experiences were obtained through interviews and questionnaires during the study visits. Age was coded as interval groups as follows: 50–54, 55–59, 60–64, 65–69, 70–74, and ≥ 75 years.

#### Cognition

Cognitive status was assessed using the subset of SPMSQ and 3 additional questions, namely 3-item, 10-item, and backward serial recalls. The subset of SPMSQ includes the following domains: (1) orientation, (2) calculation, (3) short-term memory, and (4) language. One item of SPMSQ was excluded from the analysis because it was not assessed in 2003. The total scores range from 0 to 30, with a higher score indicating more favorable cognitive status. The Cronbach’s alpha value of the 9 items of SPMSQ with recall questions in this database ranged from 0.62—0.89 for the 4 waves, indicating an acceptable internal consistency of the scale.

#### Mobility

The mobility assessment consisted of 9 self-report items on participants’ difficulty to perform the following tasks without any assistance [30, 31]: (1) 15-min standing, (2) 2-h standing, (3) squatting, (4) arm lifting overhead, (5) hand grabbing and pinching, (6) object holding (up to 12 kg), (7) running (20–30 m), (8) walking (200–300 m), and (9) stair climbing (2–3 floors). Participants rated the degree of difficulty they experienced in completing the tasks on a scale of 0 to 3 (0 = no difficulties, 1 = some difficulty, 2 = considerable difficulty, 3 = unable to complete). A summary score for mobility difficulty was used in the inferential analysis. The Cronbach’s alpha value of the 9 items ranged from 0.93—0.94 for the 4 waves in this database, indicating a good internal consistency.

#### Walking and walking device use

Walking status was determined by asking participants to rate their walking status, level of difficulty in indoor walking, and whether they use an assistive device such as a cane or a walker.

#### Depressive symptoms

The 10-item CES-D was used to screen for depressive symptoms in the study. The CES-D was developed and validated by Radloff [32] and contains questions that measures depressed mood, feelings of guilt and worthlessness, feelings of helplessness and hopelessness, psychomotor retardation, loss of appetite, and sleep disturbance. The participants rated how often they perceived depressive symptoms in the preceding week. Scores range from 0 to 30, with high scores indicating greater depressive symptoms. The instrument was commonly used for community-dwelling older population and showed adequate reliabilities (Cronbach’s alpha: 0.7—0.93) [33, 34]. One item of CES-D was excluded from the analysis because it was not assessed in 2003.

#### Engagement of leisure activities and physical activities

The scale of leisure activity engagement consisted of 9 self-report items on whether the participant engage in the following 3 types of activities: (1) passive activities: watching television, listening to radio, and reading, (2) social activities: playing chess, gathering with relatives, friends or neighbors, and group activities (3) physical activities: gardening, walking, biking, and jogging . Physical activities was determined by asking participants the duration when they perform regular exercise each time.

### Statistical analysis

Descriptive statistics including means, standard deviations, and frequencies were calculated. The variables of interest in the multivariate analysis were demographic characteristics, functional status and mobility, cognitive scores, depressive symptoms, sensory impairment, and number of comorbid illnesses. To examine whether the mobility difficulties of participants were associated with self-reported longitudinal cognitive scores, and sensory impairment, linear mixed-effects regression models were adopted. Probabilities and 95% confidence intervals were calculated for each predictor variable. The association of repeated falls with mobility, walking aid use, and living alone was examined using cumulative logit regression (SAS PROC GENMOD). PROC GENMOD enabled us to obtain models with nonnormally distributed data and specification of both time-varying (ie, fall occurrence, mobility difficulty, cognitive scores, depressive symptoms, number of comorbid illnesses, sensory impairment, and walking device use) and time-constant (ie, sex) variables.

This model tested the contributions of demographic characteristics (age group, sex, living alone), walking aid use, and cognitive scores on mobility difficulty. We controlled for the potential confounding effect of BMI, education, number of comorbid illnesses, and sensory impairment as these factors have been associated with both mobility and falls in previous studies.

Multiple imputations were used to address missing values caused by attrition. The SAS MI procedure was used to generate 10 imputed data sets based on the Markov chain Monte Carlo method. Analysis results for the 10 imputed data sets were combined using PROC MIANALYSE. We used SAS 9.4 (SAS Institute, Cary, NC, USA) to complete all analyses.

## Results

### Participant characteristics

In 2003, the total number of participants was 5267, of which 2696 were men and 2571 were women, yielding a male-to-female ratio of 1.05. Approximately 49.5% of the participants were younger than 65 years at baseline. Among the participants, 1933 died during the study period (593 between 2003 and 2007; 727 between 2007 and 2011; 613 between 2011 and 2015). In 2015, 2890 participants completed the follow-up interview.

Table 1 presents the demographic and health characteristics of the participants at baseline in 2003. Approximately 5.16% of the individuals reported having indoor walking difficulties, and 47 (17.28%) of them used an assistive device to walk.

**Table 1:** Demographic and health characteristics at baseline

	2003 (*n* = 5267)	
	mean/frequency	SD / %
Sex (Male)	2696	51.19
Age group 50–54	1237	23.49%
55–59	684	12.99%
60–64	687	13.04%
65–69	567	10.77%
70–74	580	11.01%
75 +	1512	28.71%
Cognitive score	20.41	4.89
(0–30)		
Mobility difficulty	4.46	6.65
(0–27)		
CES-D	4.78	5.54
(0–30)		
Number of comorbid illnesses	1.60	1.57
(0–11)		
Living alone	415	7.88%
Indoor walking difficulty		
0 (no difficulty)	4995	94.84%
1 (some difficulties)	110	2.09%
2 (considerable difficulties)	48	0.91%
3 (unable to complete)	114	2.16%

*Abbreviation*: *SD* Standard deviation, *CES-D* Center for epidemiological studies depression

### Mobility difficulty and personal characteristics

Table 2 summarizes the parameters resulting from the linear mixed-effects regression models. For the entire cohort, with the 50–54 age group as the reference group, mobility difficulties were pronounced in the 60–64 age group. Mixed regression modeling indicated that having lower cognitive scores (b =  − 0.14, *p* = .0326) was associated with a greater rate of mobility difficulty. Female sex (b = 1.25, *p* < .0001), greater number of comorbid illnesses (b = 0.51, *p* = .0017), lower BMI (b =  − 0.07, *p* = .0044), and hearing loss (b = 0.73, *p* = .0493) also had an increased levels of mobility difficulty. Engagement with leisure activities is also negatively associated with mobility difficulty: passive activities (watching television, listening to radio, and reading) (b =  − 0.86, *p* = .0031), social activities (playing chess, gathering with relatives, friends or neighbors, and group activities) (b =  − 0.80, *p* = .0061), and physical activities (gardening, walking, biking, and jogging) (b =  − 0.83, *p* = .0009). The longer workout duration also suggests slower rate of mobility difficulty (b =  − 0.66, *p* = .0200).

**Table 2:** Unstandardized parameters resulting from linear mixed effects regression models predicting mobility difficulties (*n* = 5267)

Fixed effects	Estimate (SE)	Confidence limits. (lower, upper)	*p*-value
Intercept	6.78 (2.59)	0.97, 12.59	*p* = *.0267*
Year 4-year	0.92 (0.15)	0.60, 1.24	*p* < *.0001*
8-year	2.25 (0.23)	1.75, 2.74	*p* < *.0001*
12-year	2.93 (0.27)	2.34, 3.53	*p* < *.0001*
Age 50–54 (ref)			
55–59	0.14 (0.21)	-0.28, 0.55	*p* = *.5239*
60–64	0.64 (0.24)	0.17, 1.12	*p* = *.0082*
65–69	2.03 (0.28)	1.47, 2.59	*p* < *.0001*
70–74	3.42 (0.31)	2.79, 4.04	*p* < *.0001*
75 +	5.22 (0.37)	4.43, 6.00	*p* < *.0001*
Sex (Female)	1.25 (0.15)	0.95, 1.56	*p* < *.0001*
Number of comorbid illnesses	0.51 (0.12)	0.25, 0.78	*p* = *.0017*
CES-D	0.19 (0.04)	0.10, 0.29	*p* = *.0009*
Cognitive score	-0.14 (0.05)	-0.26, -0.01	*p* = *.0326*
Passive leisure acts	-0.86 (0.22)	-1.34, -0.36	*p* = *.0031*
Social leisure acts	-0.80 (0.23)	-1.32, -0.28	*p* = *.0061*
Physical leisure acts	-0.83 (0.18)	-1.24, -0.43	*p* = *.0009*
Workout duration	-0.66 (0.23)	-1.19, -0.13	*p* = *.0200*
Hearing loss	0.73 (0.32)	0.00, 1.46	*p* = *.0493*
Visual impairment	0.24 (0.27)	-0.37, 0.86	*p* = *.3963*
BMI	-0.07 (0.02)	-0.11, -0.02	*p* = *.0044*

*Abbreviation*: acts activities, *CES-D* Center for epidemiological studies depression, *SE* Standard error

**Fig. 1:**
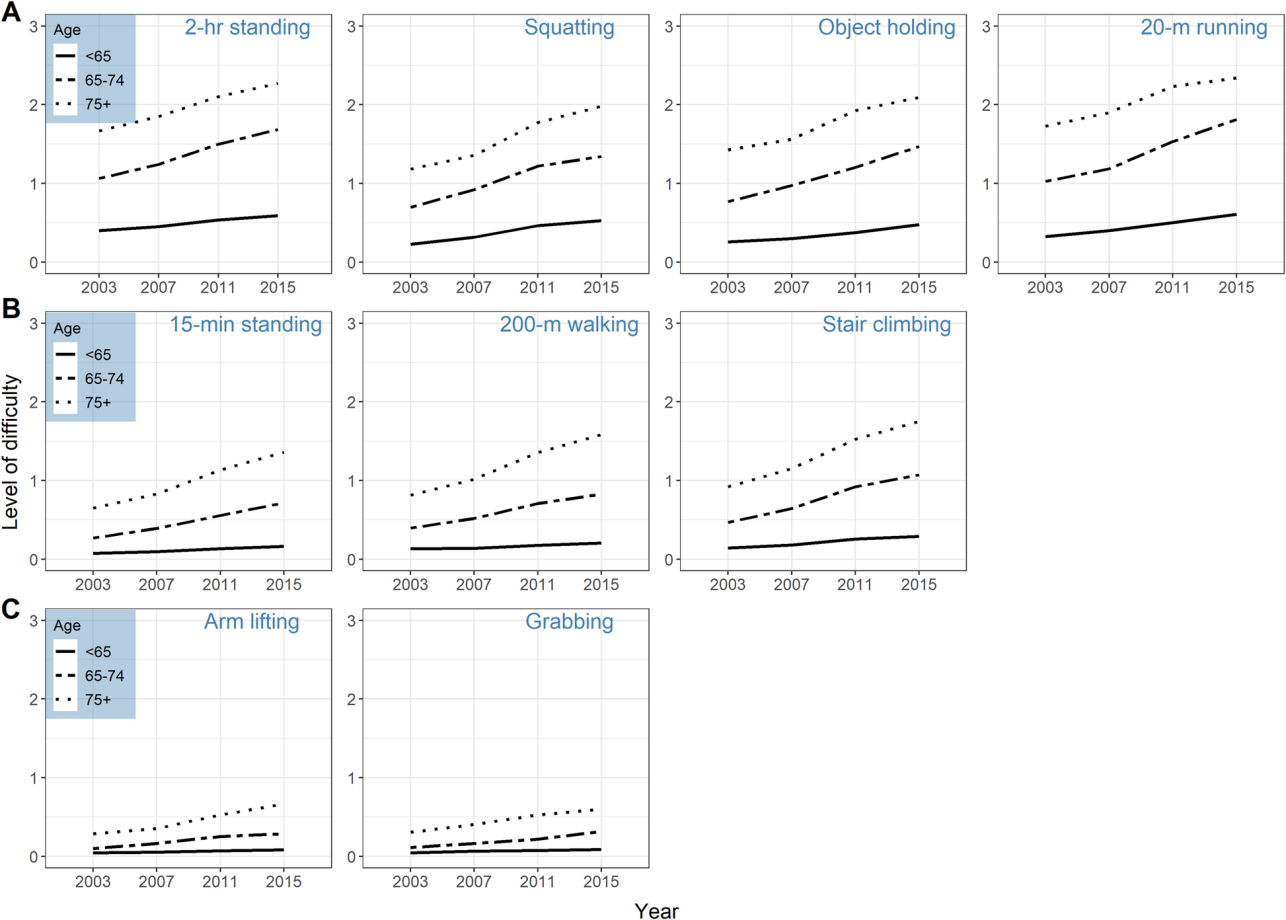
The trajectory of perceived difficulty in performing each mobility task over time. Age was regrouped into 3 categories in this figure: < 65 years (pre-old), 65-74 years (old), and > 75 years (old-old).

The levels of difficulty in performing each mobility task were analyzed to assess how they associate with the personal and health factors. Three distinct groups of tasks were identified according to the age at which participants began to perceive difficulties, namely (1) Group A, difficulty starting from age 60: 2-h standing (b = 0.20, *p* < .0001), squatting (b = 0.14, *p* = .0001), object holding (b = 0.10, *p* = .0033), and running (b = 0.21, p < .0001); (2) Group B, difficulty starting from age 65: 15-min standing (b = 0.09, *p* = .02), walking (b = 0.14, *p* = .0004), and stair climbing (b = 0.21, *p* < .0001); (3) Group C, difficulty starting from age 75 or with no change: grabbing (b = 0.10, *p* = .04) and arm lifting (b = 0.08, *p* = .10). Figure 1 presents the grouping of the mobility tasks by age and the trajectory of the perceived difficulty over time. Difficulties in running (b =  − 0.03, *p* < .0001), stair climbing (b =  − 0.02, *p* < .0001), 2-h standing (b =  − 0.03, *p* < .0001), squatting (b =  − 0.02, *p* = .0013), and object holding (b =  − 0.02, *p* < .0001) were associated with lower cognitive scores. Walking, 15-min standing, grabbing and arm lifting did not have significant associations with cognitive scores. The detailed information is shown in the Appendix B.

### Falls and assistive walking devices

Table 3 summarizes the fall occurrence and repeated falls (fell 2 times or more) of the study. Self-reported fall occurrence in the preceding year at baseline (first visit) was 15.76%, 19.98% at the second visit, 21.12% at the third visit, and 20.62% at the fourth visit. The data revealed that 10.28% of the population aged 50–64 years and 21.24% of those aged ≥ 65 years fell in the year preceding the baseline interview. During the 12-year study with 4 visits, up to 45% of walking device users perceived their walking status as poor or very poor.

**Table 3:** Fall occurrence, repeated falls, and walking device use

Main group: Fall occurrence								
	2003		2007		2011		2015	
	*n* = 5267		*n* = 4330		*n* = 3579		*n* = 2890	
	Non-faller	Faller	Non-faller	Faller	Non-faller	Faller	Non-faller	Faller
	4437 (84.2%)	830 (15.8%)	3465 (80.0%)	865 (20.0%)	2823 (78.9%)	756 (21.1%)	2293 (79.3%)	596 (20.6%)
Gender (n)								
Male	2360	336	1794	375	1423	326	1151	256
Female	2077	494	1671	490	1400	430	1142	340
Age (n)								
50–54	1133	104	951	130	861	147	796	135
55–59	606	78	506	98	468	90	407	101
60–64	601	86	490	118	450	111	388	87
65–69	492	75	404	102	313	112	243	112
70–74	460	120	368	108	274	93	192	72
75 +	1145	367	746	309	457	203	267	89
Repeated falls (n, %)	–	385, 7.0%	–	314, 7.2%	–	337 20.6%	–	248, 7.6%
	2003		2007		2011		2015	
Walking device users	*n* = 559		*n* = 587		*n* = 617		*n* = 511	

Table 4 presents the logit estimates for repeated falls (2 times or more) relative to the reference category. We observed a significant association of falls with follow-up time, age, cognitive scores, depressive symptoms, and number of comorbid illnesses with follow-up time exhibiting the strongest association with fall occurrence. Overall, the predictors of fall occurrence that were included in the risk profile were greater mobility difficulty (b = 0.06, *p* < .0001), more depressive symptoms (b = 0.02, *p* = .0054), higher number of comorbid illness (b = 0.08, *p* = .0009), living alone (b = 0.42, *p* = .0011), and walking device use (b = 1.45, *p* = .0027). Follow-up time was a significant factor for mobility difficulty and falls. No associations were noted for sex, visual impairment, or hearing loss.

**Table 4:** Unstandardized parameters resulting from cumulative logit models for repeated falls (*n* = 5267)

	Estimate (SE)	Confidence limits (Lower, upper)	*p*-value
Intercept 1	-2.94 (0.22)	-3.41, -2.48	*p* < *.0001*
Intercept 2	-2.00 (0.22)	-2.45, -1.54	*p* < .0001
Year			
4-year	0.61 (0.05)	0.50, 0.71	*p < .0001*
8-year	0.81 (0.07)	0.68, 0.94	*p* < .0001
12-year	0.85 (0.07)	0.72, 0.99	*p* < .0001
Age 50–54 (ref)			
55–59	0.09 (0.07)	-0.05, 0.22	*p* = .2212
60–64	0.03 (0.08)	-0.12, 0.18	*p* = .6899
65–69	0.09 (0.09)	-0.08, 0.26	*p* = .2911
70–74	0.24 (0.08)	0.09, 0.39	*p* = .0016
75 +	0.35 (0.08)	0.17, 0.52	*p* = .0003
Cognitive score	-0.02 (0.01)	-0.03, -0.01	*p* = .0081
CES-D	0.02 (0.01)	0.01, 0.03	*p* = .0054
Hearing loss	0.03 (0.04)	-0.05, 0.11	*p* = .4213
Visual impairment	0.01 (0.03)	-0.06, 0.08	*p* = .7490
Mobility difficulty	0.06 (0.00)	0.05, 0.07	*p* < .0001
Number of comorbid illnesses	0.08 (0.02)	0.04, 0.12	*p* = .0009
Alone	0.42 (0.10)	0.19, 0.64	*p* = .0011
Passive leisure act	-0.04 (0.04)	-0.13, 0.04	*p* = .3093
Social leisure act	0.03 (0.04)	-0.06, 0.12	*p* = .4784
Physical leisure act	-0.02 (0.03)	-0.09, 0.04	*p* = .4560
Workout duration	-0.06 (0.02)	-0.11, -0.02	*p* = .0113
Use walking device (UWD)	1.45 (0.43)	0.56, 2.34	*p* = .0027
Indoor walking difficulty no difficulty (ref)			
some difficulties	0.84 (0.20)	0.42, 1.25	*p* = .0003
considerable difficulties	-0.37 (0.28)	-0.92, 0.17	*p* = .1768
unable to complete	-1.74 (0.16)	-2.06, -1.43	*p* < .0001
Interaction term: Indoor walking difficulty × UWD			
Some difficulties × UWD	-1.26 (0.43)	-2.14, -0.38	*p* = .0062
Considerable difficulties × UWD	-0.71 (0.46)	-1.62, 0.20	*p* = .1231
Unable to complete × UWD	0.10 (0.45)	-0.83, 1.03	*p* = .8226

*Abbreviation*: *acts* activities, *CES-D* Center for epidemiological studies depression, *SE* Standard error, *UWD* Use walking device

We used cumulative logit models to assess whether the interaction of walking device use and indoor walking difficulty was associated with repeated falls. After controlling for age, we observed that the probability of repeated falls was associated with greater mobility difficulty, cognitive score, hearing loss, depressive symptoms, shorter duration of physical activity and living alone. Moreover, although walking device use increased the probability of repeated falls, the probability of repeated falls was significantly reduced in those with some walking difficulties and who use a walking device (interaction term: indoor walking difficulty × walking device use, b =  − 1.26, *p* = .0062).

## Discussion

This study demonstrates the risk factors associated with mobility difficulty in a community-based population of middle-aged and older adults who were followed up for 12 years. The main findings are as follows: (1) longitudinal appearance of mobility difficulty was significantly associated with changes in cognitive score, number of illnesses, and hearing loss, starting from 60 years old. The acceleration of the perceived difficulty in each mobility task was distinctive. (2) The older population had the most risk factors for repeated falls, with strong associations noted for lower cognitive scores, living alone, and hearing loss. Walking device use in individuals with some walking difficulties could help lower the likelihood of repeated falls. (3) Engagement of physical or leisure activities in daily life could help to reduce the risk of mobility difficulty and falls.

Studies have indicated that mobility difficulty are a function of age in older adults. A widely held notion is that appearance of mobility difficulty and falls only occur in older people. However, in the TLSA study, marked increases in mobility difficulties have been noted in participants from the age of 60 years. Consistent with the results of related studies, we observed that mobility difficulties were greater in women than in men as people age. The mobility change over time suggests that functional deterioration is a critical issue in later life. This finding supports the idea that mobility difficulty signals greater vulnerability to adverse health outcomes in later life.

Some differences in the trajectories of reported mobility difficulty should be noted. Overall, the participants’ mobility dropped substantially from age 60. In terms of mobility tasks, difficulty in grabbing and arm lifting emerged mostly at an older age (75 + years), whereas difficulties in squatting, short-distance running, 2-h standing, and object holding were already noted in those aged ≥ 60 years. As people age, mobility in the lower extremities starts deteriorating. This deterioration indicates that adults may experience greater movement limitations from middle age to old age. Hand functions such as gripping or pinching are controlled by the musculoskeletal and nervous systems and are crucial for daily tasks such as driving or holding the walker; however, these functions begin to deteriorate after age 65 [35, 36]. Mobility tasks become demanding as ageing is associated with declines in skeletal muscle mass and aerobic capacity [37].

Mobility promotion initiatives should focus proactively on the lower extremities to help older adults maintain key functions such standing and walking. Multicomponent exercises involving aerobics, strengthening, progressive resistance, balancing, and dancing have been evidenced to improve balance and reduce the risk of falling for older adults [38]. Our finding also indicated that individuals with more frequent leisure activities including passive, social and physical activities, and longer duration of physical activity in daily life may have lower rates of mobility difficulties and repeated falls. In order to bridge prepared retiring population swiftly to community-based care stations where exercising programs were provided for adults. It is significant to be aware that the ongoing long-term care services or other accessible healthcare resources usually set 65 years of age as the eligibility criteria in Taiwan [39]. Therefore, older adults aged between 60 to 64 who might start experiencing the impacts of physical degeneration due to mobility change may have less opportunities to use the healthcare resources, especially when the early onset of motor degeneration is mild and unnoticeable.

Repeated falls are common in the older population [40, 41]. To date, no single factor has proven to be an adequate surrogate for predicting fall occurrences. Our study reveals a pronounced increase in fall occurrence in older people. Older adults were nearly twice as likely as middle-aged adults to report falls at baseline (21% vs 10.28%). Additionally, the cumulative logistic regression results indicated a higher likelihood of repeated falls among individuals older than 70 years, living alone, engaging shorter duration of physical activities, and reporting greater depressive symptoms. The literature provides evidence on increased falls of older adults living alone could experience greater social isolation and depression, which may lead to faster physical degeneration and falls [42]. Studies demonstrate group-based activity programs were helpful for older adults by increasing physical activities, reducing falls and social isolation [42–44]. Given that we found a higher likelihood of repeated falls among the 70 + year olds, living alone, reporting greater depressive symptoms, and fewer physical activity engagement, providing skills and opportunities in social and physical activity for community-dwelling population could be considered in future care plan.

Several studies have argued that walking device use is likely to result in more falls. In this study, walking device use was linked to a greater probability of falls among middle-aged and older adults. However, this does not suggest that walking device use increases falls. We also found that walking device use can significantly reduce the probability of repeated falls in those with some walking difficulties. Walking device use may involve greater cognitive demands in terms of attention or working memory, for example [45]. The risk factors for repeated falls in community-dwelling middle-aged and older adults were preexisting mobility impairment, lower cognitive score, increased number of comorbid illnesses, older age, and assistive device use. The risk of mobility decline was greater when a participant used an assistive device, implying that a more severe mobility impairment increases the likelihood of the loss of independence. When care workers and clinicians observe moderate difficulty in walking, they can employ simple questions regarding the use of any assistive walking device to identify individuals who are at risk for falls and loss of walking independence and who may benefit from preventive and rehabilitative efforts designed to maintain ambulation in community.

Changes in cognitive function are thought to be fundamental to the aging process and interact with physical functioning. In this study, cognitive scores were significantly associated with the probability of repeated falls. Interventions for high-risk older adults should include cognitive training and specific programs to improve walking safety. Because health-care providers regard falls as a major problem among older adults, support for walking safety should be strengthened, especially for walking device users and those living alone. Interventions such as walking promotion, environmental adjustment, safety checks, and escorted walking are required. The present findings may be useful in the design of further mobility support and fall prevention programs. Close observation and health promotion related to the walking function of individuals should be initiated for fall-prone participants.

The strengths of this analysis include a large cohort, a 12-year follow-up, a population-based community sample, and well-designed surveys. We conducted longitudinal data analyses to elucidate the association of personal characteristics with longitudinal appearance of mobility difficulty and falls in middle-aged and older-adult groups. However, our study was limited by the choice of assessment items, the nonassessment of some items during some years, and the lack of objective measurements. Because of the limitation of secondary data analysis, we could also not examine the environmental factors that may explain increases in mobility difficulty and falls. Future studies should incorporate biomarkers as well as environmental factors to provide more comprehensive information on the predictors of mobility difficulty and falls. The models obtained in this study are associative and cannot determine causality, since we cannot determine whether mobility difficulty precedes alterations in fall occurrence or the other way around. However, our approach provides insights into how mobility difficulty evolves by assessing the data on personal characteristics over time. More longitudinal studies are necessary to understand the nature of mobility change in community-dwelling adults. Future studies using difference cutoffs with a broader definition of mobility are also needed.

## Conclusion

The present results suggest that the personal characteristics of middle-aged and older adults may contribute to mobility difficulty and falls. Given that falls occur more often in those living alone or who use a walking device, prevention efforts should focus on increasing assistance with ambulation and providing supervised or scheduled walking.

## Supplementary Information

**Additional file 1:** **Appendix A.** Construction of TLSA sample. **Appendix B.** Unstandardized parameters resulting from linear mixed effects regression models predicting each mobility task (*n* = 5267).

## Data Availability

The data that support the findings of this study are available from the Ministry of Health and Welfare, Taiwan, but restrictions apply to the availability of these data, which were used under license for the current study, and so are not publicly available. Data are however available from the corresponding author (flkuo@nhri.edu.tw) upon reasonable request and with permission of the Ministry of Health and Welfare in Taiwan.

## Abbreviations

TLSA: Taiwan longitudinal study on aging
SPMSQ: Short portable mental status questionnaire
CES-D: Center for epidemiological studies-depression
BMI: Body mass index
SD: Standard deviation
SE: Standard error
UWD: Use walking device
