# Supplementary material for: Trajectories of mobility difficulty and falls in community-dwelling adults aged 50 + in Taiwan from 2003 to 2015
Source: BMC Geriatr. 2022 Nov 25;22:902. doi: 10.1186/s12877-022-03613-3 (PMC9700940; doi:10.1186/s12877-022-03613-3)
Supplement: Supplementary file 1 — Additional file 1: Appendix A. Construction of TLSA sample. Appendix B. Unstandardized parameters resulting from linear mixed effects regression models predicting each mobility task (n = 5267). [file 12877_2022_3613_MOESM1_ESM.docx]

Appendix

Appendix A. Construction of TLSA sample.

2003

n = 5267

2015
n = 2890

2007

n = 4330

2011

n = 3579

Deceased: 593

Lost to follow up: 344

Deceased: 727

Lost to follow up: 24

Deceased: 613

Lost to follow up: 76

Appendix B. Unstandardized parameters resulting from linear mixed effects regression models predicting each mobility task (n = 5267)

|  |  | 2-hr standing | Squatting | Object holding | 20-m running | 15-min standing | 200-m walking | Stair climbing | Arm lifting | Grabbing |
| --- | --- | --- | --- | --- | --- | --- | --- | --- | --- | --- |
| *Fixed effects* |  | Est. (SE) | Est. (SE) | Est. (SE) | Est. (SE) | Est. (SE) | Est. (SE) | Est. (SE) | Est. (SE) | Est. (SE) |
| Intercept |  | 0.97 (0.34)* | 0.29 (0.30) | 0.97 (0.36)* | 0.85 (0.32)* | 0.74 (0.36) | 0.73 (0.39) | 0.75 (0.34) | 0.55 (0.22)* | 0.48 (0.25) |
| Year 4-year |  | 0.11 (0.02)*** | 0.15 (0.02)*** | 0.10 (0.02)*** | 0.11 (0.02)** | 0.10 (0.02)*** | 0.10 (0.02)*** | 0.14 (0.02)*** | 0.04 (0.02) | 0.05 (0.02)* |
| 8-year |  | 0.22 (0.04)*** | 0.36 (0.04)*** | 0.27 (0.03)*** | 0.28 (0.03)*** | 0.25 (0.03)*** | 0.24 (0.03)*** | 0.35 (0.03)*** | 0.12 (0.03)*** | 0.10 (0.02)*** |
| 12-year |  | 0.27 (0.03)*** | 0.43 (0.04)*** | 0.38 (0.04)*** | 0.35 (0.05)*** | 0.40 (0.04)*** | 0.33 (0.04)*** | 0.41 (0.04)*** | 0.14 (0.03)*** | 0.14 (0.03)*** |
| Age 50-54 (ref) |  |  |  |  |  |  |  |  |  |  |
| 55-59 |  | 0.06 (0.03) | 0.05 (0.03) | 0.01 (0.04) | 0.05 (0.03) | -0.02 (0.03) | -0.00 (0.03) | 0.01 (0.03) | -0.02 (0.02) | -0.02 (0.02) |
| 60-64 |  | 0.19 (0.04)*** | 0.14 (0.04)*** | 0.13 (0.04)** | 0.19 (0.04)*** | -0.02 (0.03) | 0.01 (0.03) | 0.06 (0.03) | -0.07 (0.03)* | -0.05  (0.03)* |
| 65-69 |  | 0.46 (0.05)*** | 0.35 (0.04)*** | 0.34 (0.05)*** | 0.47 (0.05)*** | 0.07 (0.03)* | 0.11 (0.04)** | 0.22 (0.04)*** | -0.07 (0.03)* | -0.05 (0.03) |
| 70-74 |  | 0.61 (0.06)*** | 0.48 (0.05)*** | 0.58 (0.05)*** | 0.68 (0.05)*** | 0.22 (0.04)*** | 0.30 (0.05)*** | 0.39 (0.04)*** | -0.00 (0.04) | -0.00 (0.04) |
| 75+ |  | 0.81 (0.07)*** | 0.69 (0.05)*** | 0.83 (0.05)*** | 0.92 (0.07)*** | 0.43 (0.04)*** | 0.53 (0.05)*** | 0.61 (0.05)*** | 0.08 (0.04) | 0.10 (0.04)* |
| Sex (Female) |  | 0.16 (0.03)*** | 0.22 (0.03)*** | 0.40 (0.03)*** | 0.21 (0.03)*** | 0.04 (0.02) | 0.08 (0.02)** | 0.11 (0.02)*** | 0.01 (0.02) | 0.01 (0.02) |
| Comorbidity |  | 0.10 (0.03)** | 0.09 (0.02)** | 0.07 (0.02)* | 0.10 (0.02)*** | 0.03 (0.02) | 0.06 (0.02)* | 0.04 (0.02) | 0.04 (0.02)* | 0.03 (0.01)* |
| CES-D |  | 0.02 (0.00)*** | 0.02 (0.01)** | 0.03 (0.01)** | 0.02 (0.00)** | 0.02 (0.01)** | 0.03 (0.01)** | 0.03 (0.01)** | 0.02 (0.00)** | 0.02 (0.00)*** |
| ­Cognitive score |  | -0.03 (0.01)*** | -0.02 (0.01)** | -0.02 (0.01)** | -0.03 (0.01)*** | -0.01 (0.01) | -0.01 (0.01) | -0.02 (0.01)* | -0.01 (0.01) | -0.00 (0.01) |
| Passive leisure acts. |  | -0.06 (0.04) | -0.10 (0.04)* | -0.10 (0.03)* | -0.07 (0.04) | -0.11 (0.05)* | -0.12 (0.03)** | -0.12 (0.04)* | -0.10 (0.03)** | -0.10 (0.02)*** |
| Social leisure acts. |  | -0.09 (0.04)* | -0.08 (0.03)* | -0.10 (0.03)** | -0.09 (0.03)** | -0.09 (0.04) | -0.09 (0.03)* | -0.09 (0.04) | -0.07 (0.03)* | -0.09 (0.02)*** |
| Physical leisure acts. |  | -0.13 (0.03)** | -0.11 (0.02)*** | -0.11 (0.02)*** | -0.15 (0.02)*** | -0.10 (0.03)** | -0.12 (0.03)** | -0.11 (0.03)** | -0.01 (0.03) | -0.03 (0.02) |
| Workout duration |  | -0.08 (0.02)* | -0.08 (0.03)* | -0.062 (0.025)* | -0.054 (0.02)* | -0.09 (0.03)* | -0.11 (0.04)* | -0.10 (0.04)* | -0.04 (0.02)* | -0.04 (0.02) |
| Hearing loss |  | 0.12 (0.05)* | 0.09 (0.05) | 0.09 (0.05) | 0.13 (0.05)* | 0.08 (0.04) | 0.10 (0.04)* | 0.08 (0.04) | 0.05 (0.02) | 0.06 (0.03) |
| Visual impairment |  | 0.02 (0.04) | 0.02 (0.04) | 0.03 (0.03) | 0.02 (0.03) | 0.03 (0.04) | 0.02 (0.04) | 0.03 (0.04) | 0.03 (0.03) | 0.04 (0.03) |
| BMI |  | -0.00 (0.00) | 0.01 (0.00)*** | -0.01 (0.00)** | -0.00 (0.00) | -0.01 (0.00)* | -0.01 (0.00)* | -0.00 (0.00) | -0.01 (0.00)*** | -0.01 (0.00)*** |
| Abbreviation: Est., Estimate; BMI., Body Mass Index; CES-D., Center for Epidemiological Studies Depression; acts., activities; SE., Standard error  *: <.05; **: <.01; ***: < .001 | | | | | | | | | | |
